# Synthesis and Characterization of Macroinitiators Based on Polyorganophosphazenes for the Ring Opening Polymerization of *N*-Carboxyanhydrides

**DOI:** 10.3390/polym13091446

**Published:** 2021-04-29

**Authors:** Natalia Zashikhina, Marina Vasileva, Olga Perevedentseva, Irina Tarasenko, Tatiana Tennikova, Evgenia Korzhikova-Vlakh

**Affiliations:** 1 Institute of Macromolecular Compounds, Russian Academy of Sciences, Bolshoy pr. 31, 199004 St. Petersburg, Russia; nzashihina@bk.ru (N.Z.); mazarine@list.ru (M.V.); poa1307@mail.ru (O.P.); itarasenko@list.ru (I.T.); 2 St. Petersburg State Institute of Technology, Moskovsky Prospect 26, 190013 St. Petersburg, Russia; 3 Institute of Chemistry, Saint-Petersburg State University, Universitesky pr. 26, 198504 St. Petersburg, Russia; t.tennikova@spbu.ru

**Keywords:** polyorganophosphazenes, macroinitiator, hybrid copolymers, drug delivery systems

## Abstract

Among the various biocompatible amphiphilic copolymers, biodegradable ones are the most promising for the preparation of drug delivery systems since they are destroyed under physiological conditions, that, as a rule, reduce toxicity and provide controlled release of the drug. Hybrid graft-copolymers consisting of the main inorganic polyphosphazene chain and polypeptide side chains are of considerable interest for the development of delivery systems with a controlled degradation rate, since the main and side chains will have different degradation mechanisms (chemical and enzymatic hydrolysis, respectively). Variable particle degradation rate, controlled by the adjusting the composition of substituents, will allow selective delivery in vivo and controlled drug release. The present work proposes the preparation of biodegradable macroinitiators based on polyorganophosphazenes for the synthesis of hybrid copolymers. Synthesis of novel biodegradable macroinitiators based on polyorganophosphazenes was performed via macromolecular substitution of a polydichlorophosphazene chain with the sodium alcoholates, amines and amino acids. The composition of copolymers obtained was calculated using NMR. These polyorganophosphazenes bearing primary amino groups can be considered as convenient macroinitiators for the polymerization of NCA of α-amino acids in order to prepare hybrid copolymers polyphosphazene-graft-polypeptide. The developed macroinitiators were amphiphilic and self-assembled in the aqueous media into nanoparticles. Furthermore, the ability to encapsulate and release a model substance was demonstrated. In addition, the in vitro cytotoxicity of synthesized polymers was evaluated using two cell lines.

## 1. Introduction

A growing interest and intensive search for new biocompatible and biodegradable materials with tunable and controlled properties intended for biomedicine and bioengineering has led to the development and design of polyorganophosphazenes. Polyorganophosphazenes represent hybrid polymers consisting of an inorganic polyphosphazene chain and organic substituents in the side chains. Because of their unique, degradable backbone, the properties of polyorganophosphazenes greatly depend on the choice of organic substituents attached to the phosphorus atoms [1,2]. The major precursor inorganic polymer, polydichlorophosphazene, can be obtained by thermal ring opening polymerization or living cationic polymerization, and the chlorine atoms can easily undergo macromolecular substitution by a wide range of organic nucleophiles such as amines, alkoxides and amino acids [3,4,5]. It allows the creation of polyorganophosphazenes with versatile properties and tunable hydrolytic sensitivity [1,2], which represent crucial features for polymers used in biomedical applications. As a result, a wide range of stable polyorganophosphazenes can be obtained [1,6,7]. 

In general, the synthesis of polyorganophosphazenes is aimed at creating materials for tissue engineering [3], and only a few works devoted to the development of drug delivery systems were found in the current literature [8,9,10,11,12,13,14]. Polyorganophosphazenes bearing L-isoleucine ethyl ester, glycine allyl ester, and α-amino-ω-methoxy-poly(ethylene glycol) substituents were tested as delivery vehicles for the anticancer drug doxorubicin [8]. Camptothecin loaded in poly(bis(carboxyphenoxy)phosphazene)) stabilized gold nanoparticles was more efficient than free camptothecin [4]. Synthesis and characterization of polyorganophosphazenes substituted with 4-methoxybenzylamine and 4-methoxyphenethylamine to obtain indomethacin and 5-fluorouracil encapsulated forms were described by Gudasi et al. [9]. Grafted with PEG and ethyl-p-aminobenzoate, polyphosphazenes were capable of self-assembling into polymersomes, which were applied for delivery of doxorubicin hydrochloride (DOX·HCl) or co-delivery DOX with chloroquine phosphate [10,11]. Poly(phosphazene)-docetaxel conjugates were developed by grafting to the polyphosphazene backbone with multifunctional lysine ethyl ester, which acted as a spacer with methoxy poly(ethylene glycol), followed by docetaxel grafting [12]. Polyorganophosphazene-paclitaxel conjugate gels for antitumor applications have demonstrated significant antitumor activity in breast, gastric, colon, and cervix cancer cells [13]. In another study, methotrexate and gemcitabine in the form of azo prodrug were conjugated to the polyphosphazenes and used for colon cancer-specific drug delivery [14].

The way of modification and the nature of substituents affects the hydrophilicity of polyorganophosphazene and determines the biocompatibility and biodegradation rate [15]. The choice of substituents plays a crucial role, since it allows regulating physical, chemical, and biological properties of the polymer [16]. The prospective substituents to prepare hybrid polyorganophosphazenes for biomedical applications are amino acids and peptides. The diversity of amino acids allows the regulation of properties in a wide range. In turn, the degradation products of this kind of polymer are ammonia, phosphates, and a mixture of amino acids, which are easily metabolized. The sensitivity of peptides to external stimuli can be used to create materials that can change the structure, size, or other properties to obtain targeted delivery systems [17]. Moreover, synthesis of hybrid graft copolymers consisting of the main inorganic polyphosphazene chain and polypeptide side chains is of considerable interest for the development of delivery systems with a controlled degradation rate, since the main and side chains will have different degradation mechanisms (chemical and enzymatic hydrolysis, respectively).

Recently, Wilfert et al. reported the application of glycine and valine as a spacer between the polyphosphazene chain and random poly(ethylene oxide-co-propylene oxide), and observed tunable degradation rates ranging from days to months, adjusted by changes in the chemical structure of polyphosphazene [2]. Poly[(p-methylphenoxy)-(50%ethyl glycinato)phosphazene] and poly[bis(ethyl glycinato)phosphazene], synthetized by El-Amin et al. [18], appeared to support bone growth comparable to the control poly(lactide-co-glycolide). Moreover, polyorganophosphazenes containing L-alanine and L-phenylalanine, protected with alkyl esters with increasing chain length from 5 to 8 carbon atoms, were investigated as ligament and tendon tissue engineering scaffold materials [19]. The synthesis of polyorganophosphazenes containing peptides in their composition is described elsewhere [17]. In that research the peptide was introduced into the polymer structure via substitution of the chlorine atoms of polyphosphazene by the tetrapeptide Gly-Phe-Leu-Gly. Furthermore, co-substitution of such polymers with polyalkylene oxide led to the formation of water soluble and biodegradable hybrid polymers. 

The aim of this study was the synthesis of polyorganophosphazenes suitable as the macroinitiators for further polymerization of *N*-carboxyanhydrides (NCA) of α-amino acids. Ring-opening polymerization of *N*-carboxyanhydrides is the most widely applied strategy for the synthesis of polypeptides of high molecular weight with no detectable racemization at the chiral centers [20,21]. According to possible mechanisms of polymerization of NCAs, initiation with primary amines allows obtaining the well-defined polymers with narrow molecular weight distribution [22]. Thus, polyorganophosphazenes bearing primary amino groups can be considered as convenient macroinitiators for the polymerization of NCA of α-amino acids in order to prepare hybrid graft-copolymers, namely, polyphosphazene-graft-polypeptide.

In this paper, a series of various polyorganophosphazenes containing alkoxyl or amine and/or amino acid substituents with different ratios have been synthesized. The approach to their synthesis consisted of sequential synthesis of polydichlorophosphazene by thermal ring opening polymerization and substitution of the phosphorus-bound chlorine atoms with organic side groups by the reaction with alcoholates, amines and amino acids. The polyorganophosphazenes containing protected primary amino groups were prepared using bifunctional linker; they represent pro-macroinitiator for the polymerization of α-amino acid *N*-carboxyanhydrides, which can be used as a macroinitiator after deprotection. The self-assembling of synthesized copolymers was shown using the dynamic light scattering method. In addition, the ability to encapsulate and release the model substance was confirmed.

## 2. Materials and Methods

### 2.1. Materials

L-alanine ethyl ester hydrochloride (99%), L-phenylalanine ethyl ester hydrochloride (99%), glycine ethyl ester hydrochloride (99%), tert-butyl *N*-(2-aminoethyl)carbamate (*N*-Boc-ethylenediamine, ≥98.0%), triethylamine (TEA, ≥99%), rhodamine 6G (99%), and trifluoroacetic acid (TFA, 99%) were purchased from Sigma-Aldrich (Munich, Germany) and used as received. Ethanolamine (99%) was a product of ECOS-1 (Moscow, Russia). Hexachlorocyclotriphosphazene (HCP, 99%, Sigma-Aldrich, Munich, Germany) was purified by recrystallization from heptane followed by sublimation under vacuum. Solvents, such as tetrahydrofuran (THF), heptane, dimethylformamide (DMF), dimethyl sulfoxide (DMSO), benzene, methyl and ethyl alcohols (Vecton, St. Petersburg, Russia), were dried and distilled. All reactions were carried out under an atmosphere of dry argon.

Dialysis cellulose membranes with a molecular weight cut-off of 1000 (MWCO 1000, Orange Scientific, Braine-l’Alleud, Belgium) and dialysis tubes with a molecular weight cut-off of 3500 (MWCO 3500, GeBAflex Maxi, Yavne, Israel) were used to purify the polymers and to perform release experiments, respectively.

Human embryonic kidney cells HEK 293 and human lung carcinoma cells A549 were obtained from the Collection of University ITMO (St. Petersburg, Russia) and were cultivated in Dulbecco’s Modified Eagle’s Medium (BioloT, St. Petersburg, Russia) supplemented with 10% (*v*/*v*) fetal calf serum (FCS, BioloT, St. Petersburg, Russia), 50 U/mL of penicillin and 50 µg/mL of streptomycin (P/S) (PanEco, Moscow, Russia).

### 2.2. Instrumentation

The composition of synthesized polymers was determined by ^1^H and ^31^P NMR. Spectra were recorded on a Bruker AC-400 NMR spectrometer (Karlsruhe, Germany) at 25 °C using THF and DMSO-d_6_ as solvents. 

Molecular weight (*M_n_*) and dispersity (*Đ*) were established using size exclusion chromatography (SEC) (Shimadzu LC-20 Prominence, Kyoto, Japan) equipped with refractometric detector (RID 10-A). 

The hydrodynamic diameter and zeta potential of prepared nanoobjects were measured on a ZetasizerNano-ZS (Malvern, UK) at a scattering angle of 173° at 25 °C. The content of rhodamine 6G was determined by UV–Vis spectroscopy using a UV-1800 (Shimadzu, Kyoto, Japan) spectrometer at 527 nm. 

### 2.3. Synthesis of Polyorganophosphazenes

To purify hexachlorocyclotriphosphazene (HCP), it was recrystallized three times from heptane and then sublimated under vacuum at 50 °C. After that, 2 g of the monomer was placed in a Pyrex glass tube (10–15% of the tube volume), purged with argon, evacuated, and then was sealed under vacuum. The polymerization was carried out in bulk at 250 °C for 10 h. The resulting reaction mixture was dissolved in THF and then used without purification for further modification. Part of the reaction mixture was precipitated into benzene to determine the polymer yield. The yield of polydichlorophosphazene (PCP) varied in the range from 15 to 23%.

In order to obtain polyorganophosphazenes (POPh), the reactions of macromolecular substitution of chlorine atoms of PCP were carried out using *N*-Boc-ethylenediamine as well as Na alcoholates of ethanole (EtONa), methanole (MeONa) and ethanolamine (NH_2_-(CH_2_)_2_-ONa).

Alcoholates were prepared by an addition of ~0.5 eq. metallic sodium to 1 mL of alcohol and 3 mL of THF. The reaction was carried out with stirring until the sodium was completely dissolved. All processes of synthesis were conducted in an argon atmosphere.

The synthesis of polyorganophosphazene, in which both substituents were attached through an oxygen atom, was carried out by adding a prepared solution of MeONa and NH_2_-(CH_2_)_2_-ONa containing a 1.5-fold molar excess of the alcoholates mixture in relation to the number of chlorine atoms to the solution of PCP in THF. The molar ratio of alcoholates in the mixture to each other was 3:1. The reaction was carried out with stirring at room temperature for 24 h.

For the modification of polydichlorophosphazene with Boc-protected ethylenediamine and sodium ethylate, first, the solution of Boc-protected ethylenediamine (0.5 eq. to the number of chlorine atoms) and triethylamine in THF were added to the solution of polydichlorophosphazene in THF and the mixture was left under stirring for 24 h at room temperature. Finally, the solution of sodium ethylate in THF (3 eq. to the number of chlorine atoms) was added and the reaction was carried out for an additional 48 h at room temperature.

The synthesis of POPh capped with amino acids was performed using different ratios of ethyl esters of glycine (GlyOEt) and alanine (AlaOEt)/phenylalanine (PheOEt). *N*-Boc-protected ethylenediamine was introduced into the polymer structure to provide the preparation of a macroinitiator containing reactive amino groups.

The synthesis method was as follows. Triethylamine (TEA) was added to the mixture of amino acids (1.5-fold molar excess in relation to the number of chlorine atoms) in THF: 5-fold molar excess of TEA in relation to the amount of amino acids was added dropwise and the mixture was refluxed for 12 h. Triethylamine hydrochloride was removed by filtration. *N*-Boc-ethylenediamine (0.15 eq. to the number of chlorine atoms) and triethylamine (0.5 eq. to the number of chlorine atoms) in THF was added to the solution of polydichlorophosphazene in THF and the mixture was refluxed for 3 h. Both solutions were mixed and the final solution was refluxed for 10 h. The ratios of nucleophilic reagents used in the synthesis of the polyorganophosphazenes are summarized in Table 1.

The final polymers obtained were purified via dialysis (MWCO 1000) sequentially against a mixture of THF:H_2_O (50:50 *v*/*v*) for 6 h, then against water for 48 h. The resulting polymers were lyophilized. The ratio of substituents was calculated from ^1^H NMR spectra (DMSO-d_6_, 25 °C) as described in Supplementary Materials.

The analysis was carried out at 60 °C. Styragel Column, HMW6E (7.8 mm 300 mm, 15–20 m bead size) and DMF with 0.1 M LiBr or THF as eluents were applied. THF was used for analysis of PCP while DMF was used for analysis of POPh (Figure S1 of Supplementary Materials). The flow rate was 1 mL/min. Values of molecular weights and *Đ* were determined using poly(methyl methacrylate) standards with an *M_w_* range from 17,000 to 250,000 and *Đ* ≤ 1.14 (DMF) or polystyrene with an *M_w_* range from 2000 to 450,000 and *Đ* ≤ 1.15 (THF).

The removal of Boc-protective groups from the amino group of ethylenediamine was carried out by acidic hydrolysis in 30% trifluoroacetic acid in dimethylformamide. The resulting polymer was purified by dialysis (MWCO 1000) against water for 48 h. Thereafter, the polymer was lyophilized.

### 2.4. Preparation and Characterization of Nanoparticles

Polymer nanoparticles were obtained during the dialysis process from THF to THF:H_2_O (50:50 *v*/*v*), and then to water (see Section 2.3). Self-assembled nanoparticles were freeze-dried and stored at 4 °C. Before use, water was added to the weighted portion of the dry sample and the mixture was redispersed by 20 s exposure of ultrasound (UP 50H Hielscher Ultrasonics (Teltow, Germany) at the necessary concentration (1–10 mg/mL). The hydrodynamic size (D_h_), polydispersity index (PDI), and ζ-potential of the resulting nanosystems were determined by dynamic and elecrophoretic light scattering, respectively, at a particle concentration of 0.1 mg/mL. All measurements were at least triplicated. Data are presented as average ± SD. 

### 2.5. Dye Encapsulation and Release

Encapsulation of the model substance, the fluorescent dye rhodamine 6G, into polymer particles was performed as follows: the weighted portion of the POPh was preliminarily dissolved in DMSO to achieve the concentration 1 mg/mL, and the same volume of borate buffer (0.0125 M, pH 9.4) was added to the solution. Thereafter, a dye solution in methanol (c = 4 mg/mL) was added to achieve a polymer with a dye mass ratio of 5:1. The resulting mixture was sonicated for 20 s, then left overnight at 4 °C. After freeze-drying distilled water was added to the sample and the mixture was dispersed under sonication for 60 s. Unbound rhodamine 6G was removed by dialysis through a membrane with a molecular weight cut-off of 3500 for 24 h in the dark. The concentration of rhodamine 6G in dialysates was calculated using spectrophotometric data measured at 527 nm and preliminary built calibration curve. Loading content (LC) and encapsulation efficacy (EE) were calculated as described elsewhere [23].

To study the release of the encapsulated dye, 2.4 mL of NPs (c = 1.45 mg/mL) in PBS (0.01 M, pH 7.4, 0.9% NaCl) was transferred into a dialysis tube (MWCO 3500), that was placed in 35 mL PBS (0.01 M, pH 7.4 or 6.0, 0.9% NaCl) and incubated at 37 °C. At predetermined time intervals, 4 mL of external solution was removed and 4 mL of fresh PBS was added. The amount of the released rhodamine 6G was determined as described above.

### 2.6. Cell Culture Experiments

100 μL of medium, containing 8 × 10^3^ cells, was added in each well of a 96-well plate and the cells were cultivated under a humidified atmosphere with 5% CO_2_ at 37 °C. After cultivation for 24 h, the culture medium was aspirated and 100 μL (for a 24 h experiment) or 200 μL (for a 72 h experiment) fresh medium containing NPs at a concentration range of 2 to 250 μg/mL was added in each well. 

The viability of the cells was determined using an MTT assay as described elsewhere [24]. Incubation of nanoparticles with cells was then carried out. After 24 h or 72 h the medium was gently removed and 100 μL of MTT solution (1 mg/mL) in basal medium was added into each well and kept at 37 °C for 2 h. Finally, the solution was removed and formazan crystals formed by survived cells were dissolved in 100 μL DMSO for 30 min at room temperature. The quantification of viable cells was performed by measuring fluorescence intensity (λ_ex_ = 570, λ_em_ = 690 nm). Values measured at 690 nm were subtracted from the values at 570 nm for background correction. The data were calculated as a percent in relation to control wells containing untreated cells and as an average ± SD (n = 3).

## 3. Results and Discussion

### 3.1. Synthesis of Polyorganophosphazenes

The aim of this study was the synthesis of polyorganophosphazenes suitable as the macroinitiators for further polymerization of *N*-carboxyanhydrides (NCA). Ring-opening polymerization (ROP) of α-amino acids NCA is the most applied approach for the synthesis of polypeptides or their copolymers [18,19]. Using primary amines as initiators for NCA ROP allows obtaining well-defined polymers with low dispersity [20]. Therefore, polyorganophosphazenes bearing primary amino groups are suitable as the macroinitiators for further polymerization of NCA in order to prepare hybrid polyphosphazene-graft-polypeptide copolymers.

**Figure 1:**
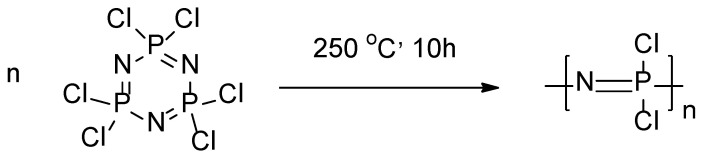
Scheme of synthesis of polydichlorophosphazene by thermal ring-opening polymerization (TROP).

A convenient method for the synthesis of polyorganophosphazenes (POPh) is nucleophilic substitution of chlorine atoms in polydichlorophosphazene (PCP). The main precursor, polydichlorophosphazene, was obtained by thermal ring-opening polymerization (TROP) of hexachlorocyclotriphosphazene (Figure 1).

It is known that this method can lead to the formation of a cross-linked polymer, which is not suitable for the synthesis of POPh due to the low conversion of P-Cl groups. This can be avoided by controlling the reaction time and temperature, since the cross-linked polymer is formed at high monomer conversions and temperatures above 250 °C [25]. Furthermore, the reaction is sensitive even to trace amounts of water and oxygen. For this reason, the reaction was carried out in pre-evacuated sealed ampoules for 10 h at 250 °C. The ^31^P spectrum of linear PCP isolated from the mixture is shown in Figure S2 of Supplementary Materials. The peak at −18.2 ppm corresponds to the P signal in unbranched PCP. The signal at 20 ppm corresponds to the phosphorus atom in hexachlorocyclotriphosphazene, trace amounts of which remained after recrystallization. This also indicates the absence of a cross-linked or hydrolyzed polymer in the mixture, the signals of which are shifted to −12.2 and −0.5 ppm, respectively [26]. According to size exclusion chromatography (SEC) data in THF, the synthetized PCPs were characterized by *M_n_* = 5900 and 8000, *Ð* = 1.04 and 1.14, respectively.

Polydichlorophosphazene is extremely hydrolytically unstable, while labile P-Cl bonds undergo nucleophilic substitution with the formation of stable polyorganophosphazenes. The choice of substituents at this stage plays a crucial role, since as mentioned above, properties of the obtained hybrid copolymers greatly depend on the choice of organic substituents associated with phosphorus atoms, as well as their ratio to each other in the polymer [27]. In this work, we synthetized polyorganophosphazenes with different possible ways of substitutions of organic molecules. Organic substituents were attached to the phosphorus atom of the main chain as follows: (1) both substituents were attached through a nitrogen atom; (2) both substituents were attached through an oxygen atom, or (3) the mixed substituents were attached forming the polymer with P-O and P-N bonds. The way how the substituent is attached to phosphorus atom and its nature affects the hydrophilicity of polymer and rate of biodegradation [13].

Ethanolamine (free or protected) or Boc-protected ethylenediamine were used to introduce the primary amino groups into the polymer structure. These organic substituents were attached to the phosphorus atom through an oxygen or nitrogen atom, respectively, that influenced their properties. The P-O bond was formed using sodium salts of various alcohols, while P-N was constructed by substitution of the chlorine atom with the amino-group in the presence of triethylamine. Boc-protected bifunctional substances were used to avoid the cross-linking of the synthetized polymers. Subsequent removal of the Boc-protecting group will lead, as in the previous case, to the formation of polymer bearing primary amino groups. As the creation of graft-copolymers did not require the grafting of each monomer unit, only 10–50% of the units were substituted with molecules, which led to the formation of (pro)carrier of NH_2_ moiety. Small methoxy or ethoxy groups were chosen as the second organic substituent, since bulky radicals can spatially block the reaction center (NH_2_). Ethyl esters of alanine/phenylalanine and glycine were used in other cases as hydrophobic and hydrophilic substituents, respectively.

**Figure 2:**
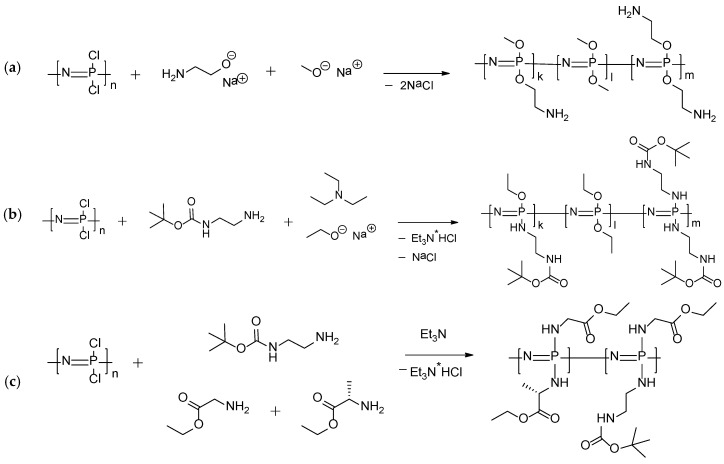

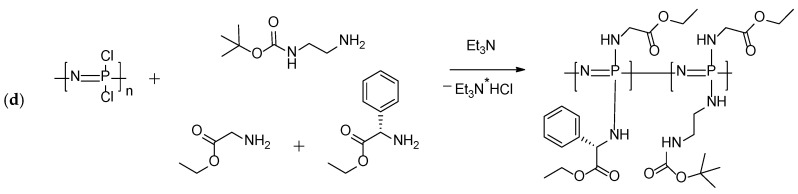
Schemes of synthesis of polyorganophospazenes containing: only P-O bonds (POPh 1) (**a**); P-O and P-N bonds (POPh-2) (**b**); or only P-N bonds (POPh 3-7) (**c**,**d**).

Thus, the series of various polyorganophospazenes with different natures and ratios of substituents have been synthesized, namely: (1) ones containing only P-O bonds (POPh 1); (2) polymers with P-O and P-N bonds (POPh 2); and (3) polymers in which both substituents were attached through a nitrogen atom (POPh 3–7). The schemes of synthesis of polymers obtained are shown in Figure 2. 

The structure of the synthesized polyorganophospazenes was confirmed by ^1^H and ^31^P NMR spectroscopy. In the ^31^P spectrum of the POPhs, in comparison to the PCP spectrum, the signal at −18 ppm disappears, which proves the completeness of the substitution reaction [25,26]. Broadened signals in the range of −20–20 ppm apparently correspond to phosphorus atoms surrounded by various combinations of substituents linked to the phosphorus atom (Figure S3). 

**Figure 3:**
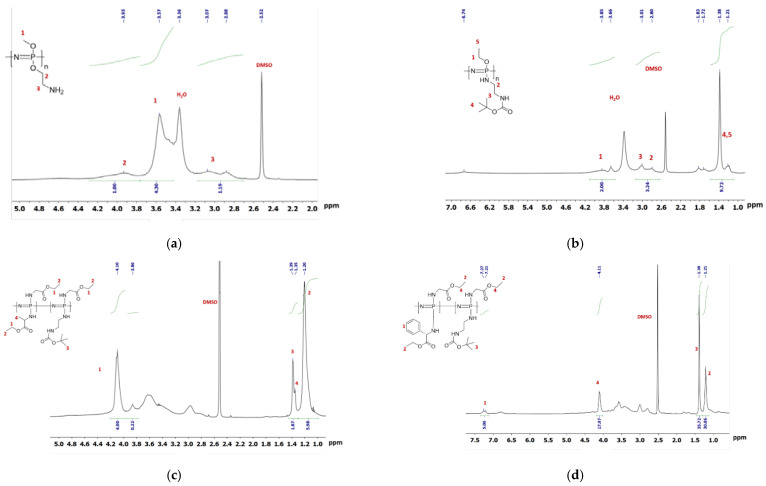
^1^H NMR spectra (DMSO-d_6_, 25 °C) of polyorganophosphazenes: POPh 1 (**a**), POPh 2 (**b**), POPh 4 (**c**), and POPh 6 (**d**).

The ratio of substituents was calculated using the relative integral areas of (a) methyl –OCH_3_ (3.57 ppm) and methylene protons (–OCH_2_–) at 3.93 ppm (POPh 1, Figure 3a); or (b) methylene protons –OCH_2_– (δ = 3.6–4.2 ppm) and –CH_2_-NH– (δ = 2.6–3.2 ppm) (POPh 2, Figure 3b); or (c) methylene protons of ethyl group (δ = 4.1 ppm) and methyl protons of ethyl group of both Gly and Ala (δ = 1.2 ppm), Boc-group (δ = 1.38 ppm) and methyl protons of Ala (POPh 4, Figure 3c); or (d) aromatic protons of Phe (δ = 7.14–7.33 ppm), methyl protons of ethyl groups of both protected amino acids (δ = 1.21 ppm) and Boc-group (δ = 1.39 ppm) (POPh 6, Figure 3d).

The ratio of substituents in the samples was determined by ^1^H NMR spectroscopy as described in Supplementary Materials. According to calculated data, the ratios of substituents in the POPh 1 are approximately equal to the composition of the initial mixture of alcoholates taken for modification. A small deviation for sample POPh 2 may be related to the steric factor. An amount of side groups with bulky substituents (–NH-CH_2_-CH_2_-NH-Boc) is lower than the initial ratio of nucleophiles in the mixture. For the samples containing amino acids, the ratio of more sterically hindered alanine/phenylalanine units in the polymer chain was significantly lower than their theoretically amount set by the ratio of nucleophiles. Thus, we can set the composition of polymers during the synthesis and vary their characteristics and properties.

The molecular weight characteristics obtained by SEC and the composition of synthesized polyorganophosphazenes are summarized in Table 2.

Deprotection of the Boc-group was carried out at acidic condition, using 30% CF_3_COOH in DMF. In the ^1^H NMR spectrum of deprotected sample POPh 4 signals of the Boc-protecting groups were decreased (Figure S4). The yield of Boc deprotection calculated from the spectra was 43%. In other words, the remaining amount of Boc-ethylenediamine was about 3% from the total amount of substituents in the polymer chain.

### 3.2. Preparation and Characterization of Nanoparticles

**Figure 4:**
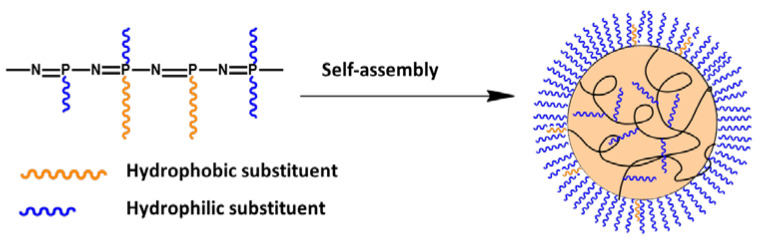
Scheme of self-assembly of amphiphilic polyorganophosphazenes.

Purification of polymers from low molecular weight impurities and the formation of nanoparticles were achieved by gradient solvent inversion from organic solvent (THF or DMF) to water. The polymers obtained were capable to self-assembly in aqueous media due to their amphiphilicity. In aqueous medium hydrophobic regions (the main inorganic chain of polyorganophosphazenes and hydrophobic substituents) tend to minimize the area of contact with water, while hydrophilic fragments, on the contrary, are exposed in aquous media. As a result, the particles are formed with a hydrophobic core surrounded by a hydrophilic surface (Figure 4). Due to the random position of substituents in the polymer chain, it can be assumed that such particles have a disordered architecture, in contrast to micelles or polymerosomes. In this case, a part of the hydrophilic fragments can be located inside the particle, while some hydrophobic fragments can be located near the surface of the particle.

The hydrodynamic diameter (D_h_), polydispersity index (PDI), and ζ-potential of the obtained nanosystems were determined by dynamic and elecrophoretic light scattering, respectively, at concentration of 0.1 mg/mL. The results are collected in Table 3 and Figure S5 of Supplementary Materials.

All the samples are characterized by PDI < 0.35 and hydrodynamic size smaller than 500 nm. The value of the ζ-potential of the POPh 1 sample is close to 0; this can be caused by the compensation of charges due to the presence of positively charged amino groups. An increase in the proportion of hydrophobic amino acid alanine (POPh 3–POPh 4) or phenylalanine (POPh 5–POPh 7) led to a decrease in the hydrodynamic particle size. A similar trend was observed in the case of nanospeheres based on random polypeptides [27] and probably is caused by the hydrophobic compaction of the particles during the polymer self-assembling.

### 3.3. Dye Encapsulation and Release Study

In order to study the possibility of application of particles based on synthetized polyorganophosphazenes as potential drug delivery systems, the model substance—fluorescent dye rhodamine 6G—was chosen for encapsulation and release investigation.

Rhodamine 6G is a water-soluble salt. The low encapsulation efficiency (EE) of hydrophilic substances is one of the main problems in the preparation of encapsulated forms because small molecules can easily diffuse into an aqueous environment during the carrier’s preparation. Various methods can be used to increase the EE. These methods are based on changing the solubility of the encapsulated substance, in particular, varying the pH or composition of the solvent [28].

At alkaline conditions, rhodamine 6G is deprotonated and becomes hydrophobic [29], so that it can be encapsulated in the hydrophobic core of the particle. For this reason, encapsulation was carried out in an aqueous-organic mixture, and Na-borate buffer solution, pH 9.4, was used as an aqueous medium.

**Figure 5:**
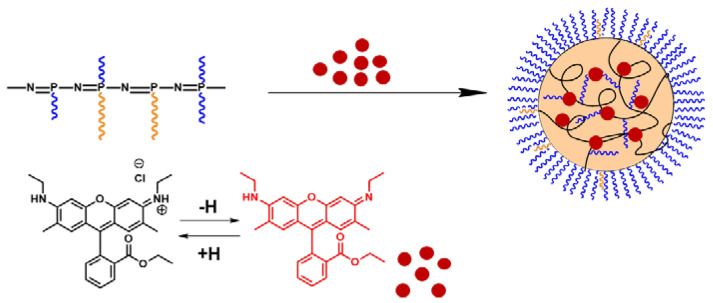
Scheme of encapsulation of Rhodamine 6G into the particles based on polyorganophosphazenes.

The encapsulation technique is shown in Figure 5. A borate buffer solution was added to the solution of dye and polymer in DMSO. The mixture was then dispersed and additionally lyophilized to increase the EE. After that, the mixture was dispersed under sonication, and the unbound dye was removed by dialysis.

The encapsulation efficiency (EE) and loading content (LC) of rhodamine into the particles based on POPh 2 were 90 ± 2%, and 180 ± 2 μg/mg of particles, respectively. Thus, the polyphosphazene hydrophobic chain promotes the incorporation of hydrophobic substances into the particles and the nanoparticles based on POPh can be considered as promising nanocarriers for encapsulation of dyes or drugs with high EE.

One of the most important characteristics of drug delivery systems is the release rate, so far as it determines a prolonged action of the encapsulated drug. The release of the dye was studied under model physiological conditions: 37 °C, 0.01 M PBS, 0.9% NaCl, pH 7.4 or 6.0, simulating the environment in the bloodstream and in the tumor microenvironment, respectively.

Dialysis was chosen as a convenient method to study the release profile [30,31]. The dye molecules released from the nanoparticles diffuse through the dialysis membrane into the external solution, from where a sample was taken for analysis.

**Figure 6:**
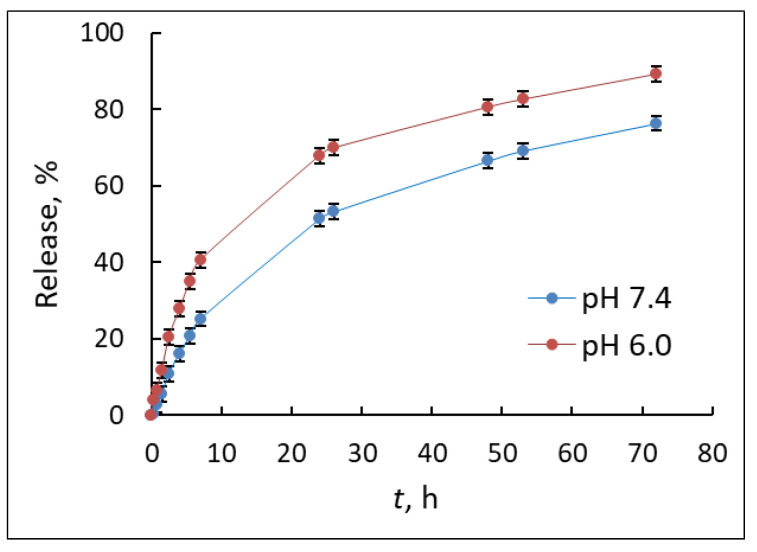
Release of model dye (rhodamine 6 G) from the particles based on POPh 2 in 0.01 M PBS with pH 7.4 and 6.0.

The release of the drug from degradable particles is a complex process, that can be explained by the diffusion of molecules through the polymer matrix and degradation of the polymer. The release profiles of rhodamine 6G at different pH are shown in Figure 6. At the first day of the experiment, the burst release was observed in both cases. During the first 8 h, 25% of the encapsulated substance was released at a pH of 7.4 and 40% at a pH of 6.0. At the same time, there is an almost linear dependence of the release on time. After 24 h of the experiment, the release rate was significantly reduced. After 72 h, 76% of the model substance was released at a pH of 7.4 and 90% at a pH of 6.0. A higher rate of rhodamine 6G release was observed with an increased acidity of the medium, which is apparently caused by protonation of the dye and, as a consequence, an increase in its hydrophilicity and solubility in the external medium.

For comparison, Ozay et al. developed phosphazene-containing microspheres and studied controlled release of rhodamine 6G from them [32]. The cumulative release for rhodamine 6G was 91.9% during 38 h in PBS. For phosphazene-tannic acid nanospheres rhodamine 6G was completely released at pH 7.0 for 12 h [33]. Hexakis [4-(acrylamido)phenoxy]cyclotriphosphazene crosslinked hydrogels were also applied for in vitro release of rhodamine 6G [34]. Cumulative release of rhodamine 6G during 30 h for media with pH 5.5 value was determined as 87%. Thus, the polyphosphazene-based particles obtained in this work can be considered as prospective systems for encapsulation of dyes or drugs.

### 3.4. Cytotoxicity Study

**Figure 7:**
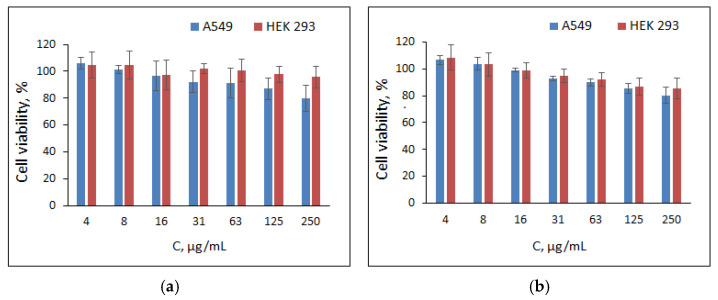
Viability of human embryonic kidney (HEK 293) and human lung carcinoma cells (A549) after their incubation for 3 days in the presence of POPh 1 (**a**) and POPh 2-based (**b**) particles.

In vitro cytotoxicity of POPh-based particles was investigated using human embryonic kidney (HEK 293) and human lung carcinoma cells (A549). Cell viability was assessed after 24 and 72 h of incubation in a medium containing the test material at various concentrations (Figure 7).

No cytotoxic effect of particles was observed at a concentration up to 250 μg/mL (viability ≥ 80%). Thus, the developed hybrid particles can be considered as potential nanocarriers for drug delivery.

## 4. Conclusions

The development of hybrid copolymers based on polyphosphazenes and polypeptides is a promising task for further creation of drug delivery systems with a controlled release of substance. In this paper, various types of polyorganophosphazenes containing primary amino groups were synthesized as macroinitiators for further ring-opening polymerization of α-amino acid *N*-carboxyanhydrides. The macroinitiators were obtained via synthesis of polydichlorophosphazene followed by controllable substitution of chlorine atoms by organic nucleophiles, including alcoholates, amines and amino acids. All synthesized macromolecules, being amphiphilic already at the stage of macroinitiators, were able to self-assembly in aqueous media into nanostructures. In addition, the self-assembled particles were found to be suitable for the entrapment of the model substance and its gradual release over 3 days. No cytotoxicity of particles was observed up to a concentration of 250 μg/mL.

The developed polyorganophosphazenes can be regarded as promising macroinitiators for further grafting of poly(amino acids) using the “grafting from” technique. In our further studies, we plan to synthesize such hybrid graft-copolymers and to study their stability, degradability and self-assembly, as well as the ability to encapsulate drugs depending on the length and nature of the side chain.

## Figures and Tables

**Table 1:** The ratios of nucleophilic reagents used in the synthesis of amphiphilic polyorganophosphazenes.

Sample	Nucleophilic Reagents 1, eqv. to Chlorine Atoms	Nucleophilic Reagents 2, eqv. to Chlorine Atoms
	[NaOCH_3_]:[NaOCH_2_CH_2_NH_2_]	
POPh 1	4.5:1.5	–
	[NH_2_–CH_2_–CH_2_–NH-Boc]	[NaO–CH_2_–CH_3_]
POPh 2	0.5	3.0
	[NH_2_–CH_2_–CH_2_–NH-Boc]	[GlyOEt]:[AlaOEt]
POPh 3	0.15	0.75:0.60
POPh 4	0.15	1.125:0.225
	[NH_2_–CH_2_–CH_2_–NH-Boc]	[GlyOEt]:[PheOEt]
POPh 5	0.15	0.75:0.60
POPh 6	0.15	0.975:0.375
POPh 7	0.15	1.20:0.23

**Table 2:** Molecular weight characteristics and composition of synthesized amphiphilic polyorganophosphazenes.

Sample	*M_n_*	*Ð*	Composition of Substituents in Final Polymer, %
			[–OCH_3_]:[–OCH_2_–CH_2_–NH_2_]
POPh 1	7700	1.14	75:25
			[–OCH_2_–CH_3_]:[–NH–CH_2_–CH_2_–NH-Boc]
POPh 2	11,300	1.16	57:43
			[–GlyOEt]:[–AlaOEt]:[–NH–CH_2_–CH_2_–NH-Boc]
POPh 3	22,200	1.35	80:13:7
POPh 4	21,200	1.32	84:10:6
			[–GlyOEt]:[–PheOEt]:[–NH–CH_2_–CH_2_–NH-Boc]
POPh 5	19,300	1.37	66:9:25
POPh 6	20,100	1.31	65:7:28
POPh 7	19,200	1.27	69:5:26

**Table 3:** Physico-chemical characteristics of self-assembled NPs (in water).

Sample	*D_h_*	*PDI*	ζ-Potential
POPh 1	430 ± 50	0.28 ± 0.02	−3 ± 1
POPh 2	240 ± 30	0.27 ± 0.06	−11 ± 2
POPh 3	90 ± 10	0.29 ± 0.01	−36 ± 2
POPh 4	105 ± 2	0.28 ± 0.02	−20 ± 3
POPh 5	160 ± 20	0.35 ± 0.06	−20 ± 1
POPh 6	190 ± 10	0.24 ± 0.01	−19 ± 2
POPh 7	230 ± 10	0.32 ± 0.01	−30 ± 2

## Data Availability

The data presented in this study are available on request from the corresponding author.

## Supplementary Materials

The following are available online at https://www.mdpi.com/article/10.3390/polym13091446/s1, Figure S1: SEC traces for POPh 3 – POPh 5 samples (in DMF); Figure S2: ^31^P NMR spectrum of PCP in THF; Figure S3: ^31^P NMR spectra of POPh 1 (a), POPh 2 (b) and POPh 4 (c) in DMSO-d_6_; Figure S4: The fragment of ^1^H NMR spectra (DMSO-d_6_) of POPh 4 (1) and one after deprotection (2). Figure S5: Size distribution by intensity measured by dynamic light scattering for POPh 2 (a) and POPh 5 (b) in water (polymer concentration—0.1 mg/mL; 3 measurements).
