# Supplementary material for: Synthesis and Characterization of Macroinitiators Based on Polyorganophosphazenes for the Ring Opening Polymerization of N-Carboxyanhydrides
Source: Polymers (Basel). 2021 Apr 29;13(9):1446. doi: 10.3390/polym13091446 (PMC8124460; doi:10.3390/polym13091446)
Supplement: Supplementary file 1 [file polymers-13-01446-s001.zip › polymers-1193413-supplementary.pdf]

polymers

MDPI

Supplementary Materials

Synthesis and Characterization of Macroinitiators Based on Polyorganophosphazenes For the Ring Opening Polymerization of N-Carboxyanhydrides

Natalia Zashikhina [1], Marina Vasileva [1], Olga Perevedentseva [1,2], Irina Tarasenko [1], Tatiana Tennikova [3] and Evgenia Korzhikova-Vlakh [1,3]

[1] Institute of Macromolecular Compounds, Russian Academy of Sciences, Bolshoy pr. 31, 199004 St. Petersburg, Russia;
[2] Saint Petersburg State Institute of Technology, Moskovsky prospect, 26, 190013, St. Petersburg, Russia;
[3] Institute of Chemistry, Saint-Petersburg State University, Universitesky pr. 26, 198504 St. Petersburg, Russia;

Analysis of ^{1}H NMR spectra

The ratio of substituents was calculated from ^{1}H NMR spectra using the relative integral areas of (1) methyl $–OCH_3$ (3.57 ppm) and methylene protons ($–OCH_2–$) at 3.93 ppm (POPh 1, Equation (1)); or (2) methylene protons $–OCH_2–$ (δ = 3.6 – 4.2 ppm) and $–CH_2$-NH– (δ = 2.6 – 3.2 ppm) (POPh 2, Equation (2)); or (3) methylene protons of the ethyl group (δ = 4.1 ppm) and methyl protons of the ethyl group of both Gly and Ala (δ = 1.2 ppm), Boc-group (δ = 1.38 ppm) and methyl protons of Ala (δ = 1.35 ppm) (POPh 4, Equations (3-4)); or (d) aromatic protons of Phe (δ = 7.14 – 7.33 ppm), methyl protons of ethyl groups of both the protected amino acids (δ = 1.21 ppm) and the Boc-group (δ = 1.39 ppm) (POPh 6, Equations (5-6)).

$$[–OCH_3] / [–OCH_2CH_2NH_2] = ((I(–OCH_3)_{3.57\ ppm}/3) / (I(–OCH_2)_{3.93\ ppm})/2)) \quad (1)$$

where $I(–OCH_3)_{3.57\ ppm}$ and $I(–OCH_2)_{3.93\ ppm}$ are relative integral areas of 3 methyl protons $–OCH_3$ at 3.57 ppm, and 2 methylene protons $–OCH_2–$ at 3.93 ppm.

$$[–O\text{-}CH_2–CH_3] / [–NH\text{-}CH_2–CH_2–NH–Boc] = ((I(–OCH_2)_{3.6\text{-}4.2\ ppm}/2) / (I(–CH_2–NH–)_{2.6\text{-}3.2\ ppm})/4)) \quad (2)$$

where $I(–OCH_2)_{3.6\text{-}4.2\ ppm}$ and $I(–CH_2–NH–)_{2.6\text{-}3.2\ ppm})$ are relative integral intensities of 2 methylene protons $–OCH_2–$ (δ = 3.6 – 4.2 ppm) and 4 methylene protons $–CH_2–NH–$ (δ = 2.6 – 3.2 ppm).

$$[–NH–CH_2–CH_2–NH–Boc] / [Ala–OEt + Gly–OEt] = (I(–C(CH_3)_{3.\ 1.39\ ppm}/9) / (I(–O–CH_2–)_{4.1\ ppm})/2) \quad (3)$$

where $I(–C(CH_3)_{3.\ 1.39\ ppm}$ and $(I(–O\text{-}CH_2–)_{4.1\ ppm}$ are relative integral intensities of 9 methyl protons of the Boc-protecting group and 2 methylene protons $–O–CH_2–$ (δ = 4.1 ppm).

$$[Ala–OEt] / [Ala–OEt + Gly–OEt] = ((I(–CH_3)_{.1.35\ ppm} / 3) / (I(–O–CH_2–)_{4.1\ ppm})/2) \quad (4)$$

where $I(–CH_3)_{.1.35\ ppm}$ and $(I(–O\text{-}CH_2–)_{4.1\ ppm}$ are relative integral intensities of the methyl protons of Ala and 2 methylene protons $–O\text{-}CH_2–$ (δ = 4.1 ppm) of both amino acids.

$$[–NH–CH_2–CH_2–NH–Boc] / [Phe–OEt + Gly–OEt] = (I(–C(CH_3)_{3.\ 1.39\ ppm}/9) / (I(–CH_3)_{1.21\ ppm})/3) \quad (5)$$

where $I(–C(CH_3)_{3.\ 1.39\ ppm}$ and $(I(–CH_3)_{1.21\ ppm})$ are relative integral intensities of 9 methyl protons of the Boc-protecting group and 3 methyl protons of the ethyl groups of both amino acids.

$$[Phe–OEt] / [Phe–OEt + Gly–OEt] = ((I(–CH_3)_{.7.14\text{-}7.33\ ppm} / 5) / (I(–CH_3–)_{1.21\ ppm})/3) \quad (6)$$

where $I(–CH_3)_{.7.14\text{-}7.33\ ppm}$ and $(I(–CH_3)_{1.21\ ppm})$ are relative integral intensities of 5 aromatic protons of the Phe methyl protons of Ala and 3 methyl protons of the ethyl groups of both amino acids.

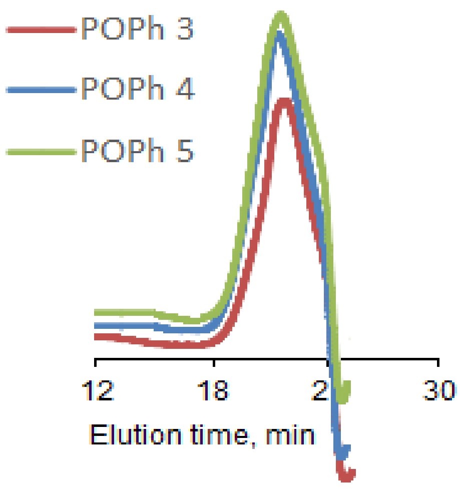

Figure S1. SEC traces for POPh 3 – POPh 5 samples (in DMF).

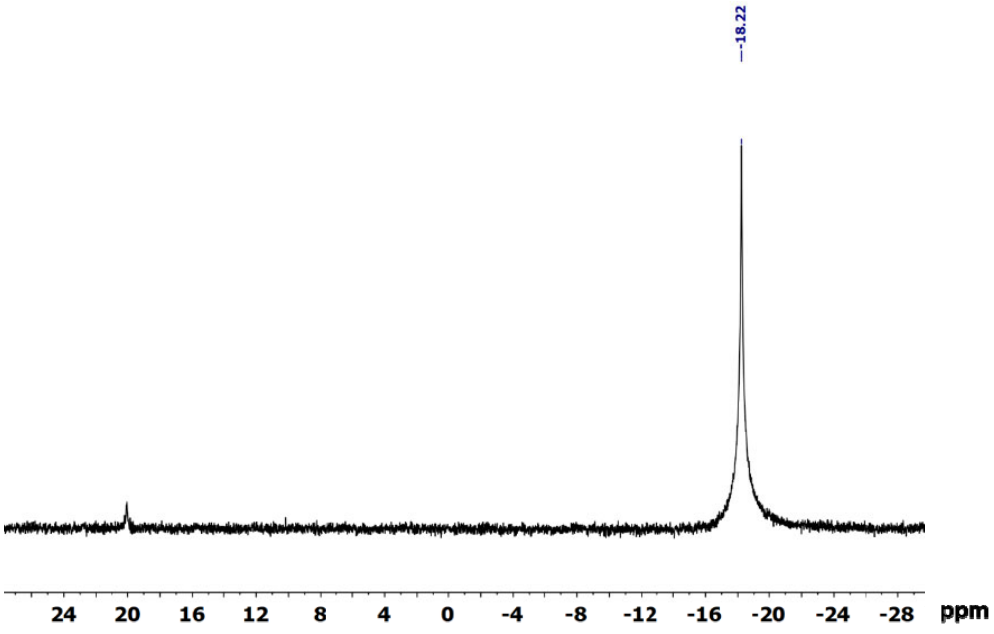

Figure S2. ^{31}P NMR spectrum of PCP in THF.

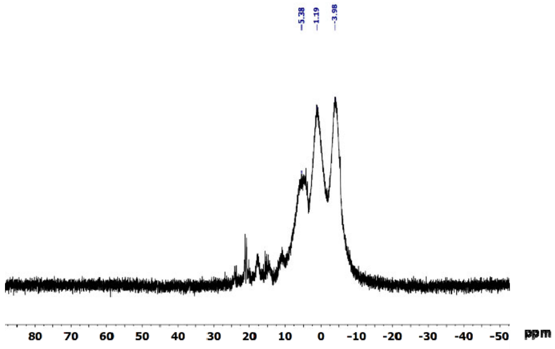

(a)

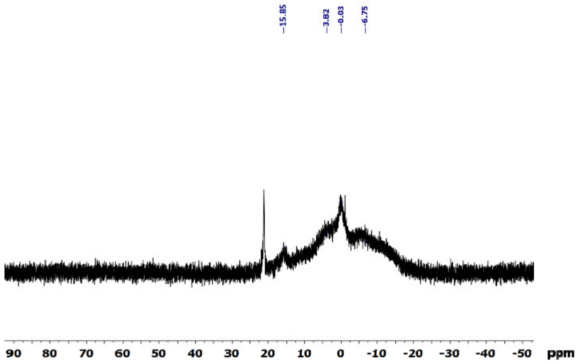

(b)

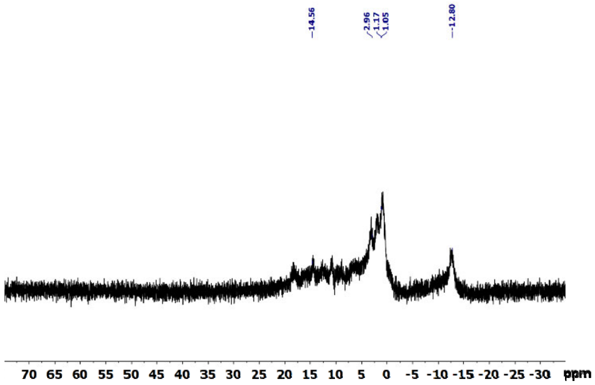

(c)

Figure S3. ^{31}P NMR spectra of POPh 1 (a), POPh 2 (b) and POPh 4 (c) in DMSO-d_6.

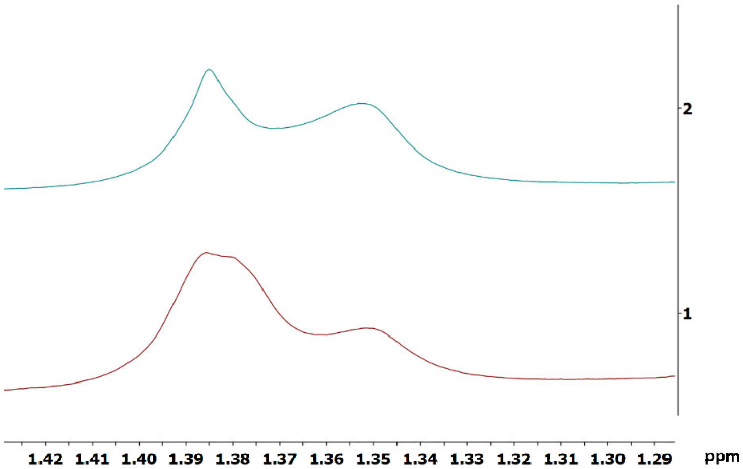

Figure S4. The fragment of ^{1}H NMR spectra (DMSO-d_6) of POPh 4 (1) and one after deprotection (2).

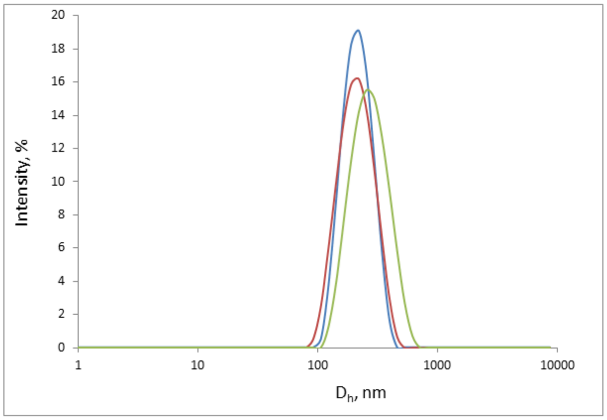

(a)

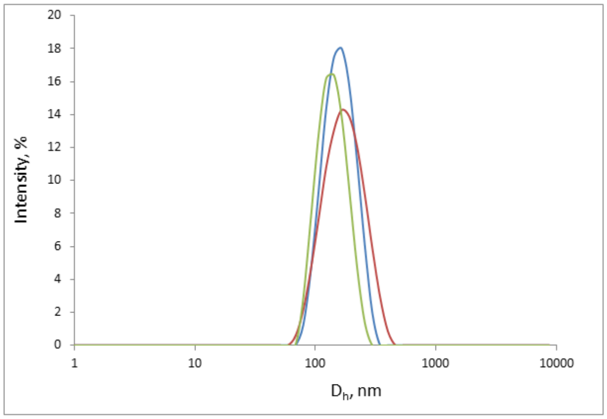

(b)

Figure S5. Size distribution by intensity measured by dynamic light scattering for POPh 2 (a) and POPh 5 (b) in water (polymer concentration—0.1 mg/mL; 3 measurements).
